# Quantification of Barbatusin and 3*β*-Hydroxy-3-deoxybarbatusin in* Plectranthus* Species by HPLC-DAD

**DOI:** 10.1155/2017/2397131

**Published:** 2017-07-03

**Authors:** Maria Goretti de Vasconcelos Silva, Leandro Bezerra Lima, Maria da Conceição Ferreira de Oliveira, Marcos Carlos de Mattos, Jair Mafezoli

**Affiliations:** Programa de Pós-Graduação em Química, Universidade Federal do Ceará, Campus do Pici, 60455-970 Fortaleza, CE, Brazil

## Abstract

The concentration of diterpenes barbatusin (**1**) and 3*β*-hydroxy-3-deoxybarbatusin (**2**) in the extracts from leaves of* Plectranthus grandis, P. barbatus, P. ornatus, *and* P. amboinicus* was evaluated by HPLC-DAD analysis on a Luna C-18 column, using isocratic mixtures of water and acetonitrile as eluents. The regression equations were obtained with good linearity (*r*^2^ > 0.99) and limit of quantifications was higher than 0.1 *µ*g/mL. The precision (lower than 3.5%, within day) and accuracy (higher than 81.7% and lower than 107.6%) of the methods were adequate. Barbatusin (**1**) was detected in* P. grandis* (15.432 ± 2.28 mg/g) and* P. barbatus* (5.198 ± 3.45 mg/g) extracts, while compound** 2 **was detected in* P. grandis *(4.068 ± 3.34 mg/g),* P. barbatus *(0.654 ± 5.86 mg/g),* P. amboinicus* (0.160 ± 7.25 mg/g), and* P. ornatus* (0.763 ± 5.10 mg/g). The evaluated validation parameters were satisfactorily achieved, and the developed methodology represents a suitable tool for application in the quantification of barbatusin (**1**) and 3*β*-hydroxy-3-deoxybarbatusin (**2**) in* Plectranthus *species.

## 1. Introduction

**Figure 1:**
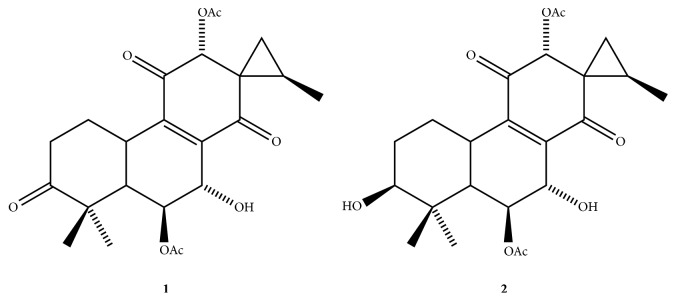
Chemical structures of barbatusin (**1**) and 3*β*-hydroxy-3-deoxybarbatusin (**2**).

The* Plectranthus* genus (Labiatae) is represented by* ca*. 300 species, which occur essentially in Africa, Asia, Australia, and some Pacific islands. Several species of this genus are cultivated for their edible tubes or as essential oil crops. In addition, some species are used for medicinal purposes such as treating vomiting, nausea, and ear infections; relieving toothache, headache, sores, and burns; or as antiseptic [1]. Species of* Plectranthus* show biosynthetic ability to produce bioactive diterpenes like the labdane diterpenoid forskolin, from* P. forskohlii*, that shows significant blood pressure lowering properties [2] and the abietane diterpenoids coleon U and horminone that show antimicrobial activity against methicillin-resistant* Staphylococcus aureus* and vancomycin-resistant* Enterococcus faecalis* [3]. In Brazil,* Plectranthus* species are popularly known as “boldo” and widely used as medicinal plants.* P. grandis* is used in the treatment of gastric and hepatic disturbs, and the gastric hyposecretory activity of this plant was already proven [4]. Several diterpenes present in this plant have shown gastroprotective properties [5–7]. These results validate the popular use of* P. grandis* in the treatment of digestive problems. The abietane diterpenes barbatusin (**1**) and 3*β*-hydroxy-deoxybarbatusin (**2**) (Figure 1) were isolated from* P. grandis *and* P. barbatus* [4, 8] and showed good gastric protection activity in rats by oral administration (10 mg/kg). The reduction of the gastric lesions was about 76% for** 1** and 96% for** 2** [8].

## 2. Experimental

### 2.1. General

Acetonitrile and methanol (HPLC grade) were purchased from Tedia®. Deionized water was obtained from a Millipore Milli-Q system. All other solvents and reagents were purchased from Synth®. Chromatography columns were developed on silica (*φ* mm 0,063–0,200) purchased from Vetec®. Analytical TLC was performed on precoated 0.25 mm thick plates of silica gel 60 F254 from Macherey-Nagel®, and the spots were visualized under UV lamp (254 nm) and by spraying with a solution of perchloric acid-vanillin in EtOH, followed by heating.

### 2.2. Plant Material

Leaves of* P. grandis *(#28377)*, P. barbatus *(#24408)*, P. ornatus *(#31929), and* P. amboinicus* (#28601), grown under environmental conditions at the Horto de Plantas Medicinais Francisco José de Abreu Matos (official medicinal plant garden from Ceará state, Brazil), were collected in January 2013, and their voucher specimens are deposited in Prisco Bezerra Herbarium-UFC.

### 2.3. LC System and Chromatographic Method

A Shimadzu UFLC chromatographic system (model LC-20A), with DAD detector (Diode Assay Detector) SPD-M20A, and LC solutions software (version 1.25) were used for data processing. The analyses were conducted on a Phenomenex® Luna 5u C18 (2) 100A (250 × 4.60 mm, 5 *µ*m) analytical column in combination with a Phenomenex guard column.

The methodology was based on the analysis of diterpenes in plants by HPLC-DAD [9]. Isocratic elution with 70% acetonitrile in water was used for compound** 1 **(method 1) and 45% acetonitrile in water for compound** 2 **(method 2). Both methods were carried out at flow rate of 0.8 mL/min. Temperature of the column was 40°C, and detection wavelength was 254 nm. Analyses were carried out in triplicate.

### 2.4. Sample Preparation and Validation

#### 2.4.1. Isolation and Identification of Compounds** 1** and** 2**

The dried leaves (3.5 Kg) of* P. grandis* were extracted with ethanol (5 × 8 L) and yielded 295.0 g of extract after solvent evaporation under reduced pressure. The crude extract was dissolved in 3 L of water/methanol (4 : 6 v/v) and submitted to liquid-liquid extraction with dichloromethane (6 × 100 mL) to yield 60.0 g of dichloromethane fraction. Successive chromatographic treatments in column, using SiO_2_ as stationary phase and dichloromethane as mobile phase, gave 3.4 g of** 1** and 140.0 mg of** 2**. The structures of both diterpenes were confirmed by high-resolution mass spectra,^ 1^H-NMR (500 MHz, CDCl_3_), ^13^C-NMR (125 MHz, CDCl_3_), and comparison with literature data [10].

#### 2.4.2. Preparation of Samples

Leaves from four* Plectranthus* species (*P. grandis, P. barbatus, P. ornatus, *and* P. amboinicus*) were dried at 40°C, pulverized to powder, and stored in a desiccator. An accurately weighted amount of 1.0 g of each sample was extracted with 100 mL of ethanol in ultrasonic bath during 1 hour at 25°C. Extracts were filtered and the solvent was completely removed by vacuum distillation at 40°C. Each plant extract was dissolved in methanol (HPLC grade) and transferred to a 25 mL volumetric flask for a final concentration 0.5 mg/mL to be analyzed by HPLC-DAD.

#### 2.4.3. Preparation of Standard Solutions

Barbatusin (**1**) and 3*β*-3-hydroxy-3-deoxybarbatusin (**2**) isolated from leaves of* P. grandis* were used as standards for the analytical curves and method validation. Each diterpene was separately dissolved in methanol (HPLC grade), and the final concentration was adjusted to 1.0 mg/mL. The purities of standard compounds** 1** and** 2** were determined as ≥99% by HPLC-DAD analyses.

#### 2.4.4. Linearity and Selectivity

Solutions of the standard compounds** 1** and** 2** were prepared in methanol at 25, 50, 100, 250, and 500 *µ*g/mL. Then, 20 *µ*L aliquots of these solutions were injected into HPLC equipment, in triplicate. For each analyte, the average peak area values were plotted against the respective concentrations, expressed in *µ*g/mL. Both the correlation coefficient (*r*) and general standard curve equation were obtained from linear least-square regression analysis (Graphpad Prism® 5.03 software). The method selectivity was determined through the analyses of the standard compounds** 1** and** 2** and extracts of the four* Plectranthus* species. In all extracts, the peaks corresponding to** 1** and** 2** were identified by comparison of their retention times with those from the standard compounds.

#### 2.4.5. Limits of Detection and Quantification

The limits of detection (LODs, S/N ≥ 3) of the two diterpenes** 1** and** 2 **were 0.015 and 0.22 *µ*g/mL, and the limits of quantification (LOQ, S/N ≥ 10) were 0.1 and 0.6 *µ*g/mL, respectively.

#### 2.4.6. Precision and Repeatability

The precision of the method was evaluated in two levels, intraday and interdays. Intraday precision was evaluated by nine determinations (triplicate) in three different concentrations. Interdays precision was evaluated using the same three concentrations, during two consecutive days, and by changing operators. The analyses were performed separately for each standard compound. The relative standard deviations (RSD) were calculated for each determination and taken as a measure of precision and repeatability.

#### 2.4.7. Accuracy

The accuracy was determined by recovery tests, which is an estimate of the accuracy of the methods. Extracts containing known amounts of** 1** (166.5, 249.0, and 374.0 *µ*g/mL) and** 2** (37.8, 47.8, and 62.8 *µ*g/mL), in quintuplicate, were analyzed, besides the extracts with no addition of standard compounds. The recoveries of both standard compounds were calculated from the corresponding calibration curve according to the following equation:

(1) $$\begin{array}{c} \%\;\;recovery=\left(\;\frac{amount\;\;found}{amount\;\;added}\;\right)\times 100. \end{array}$$

## 3. Results and Discussion

**Figure 2:**
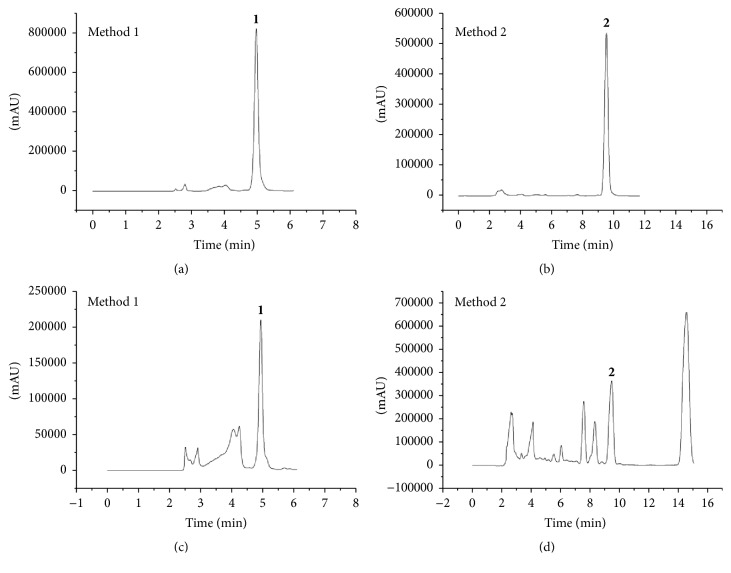
HPLC chromatograms (254 nm) of standard compounds** 1** (a) and** 2** (b) and the ethanol extract of* P. grandis* (c and d).

Two analytical curves were obtained, one for each standard compound** 1** (method 1) and compound** 2** (method 2). The linearity of the concentrations versus peak area plot for both methods was determined, and good correlation coefficients were achieved (*r*^2^ = 0.9931 and *r*^2^ = 0.9966).  Table 1 shows the linear regression equation and values for the LOD and LOQ which indicates that the developed methods are very sensitive and adequate for the investigated samples. The chromatograms of** 1** and** 2** and* P. grandis* extract are shown in Figure 2.

The intraday precision was evaluated by triplicate injection at low, medium, and high concentrations levels. Relative standard deviations were calculated based on observed concentrations. Naturally, interdays precision shows relative standard deviation greater than intraday. The accuracy was performed as recommended, by collecting data from a minimum of nine determinations over a minimum of three concentration levels [11].  Table 2 shows the precision of the methods for** 1** and** 2**, which were accurate with recovery rates ranging from 81.7 to 104.0% for** 1** and from 102.1 to 107.6% for** 2**. The found recovery rates (Table 2) were acceptable since the objective of this study is mainly estimating the amount of diterpenes** 1** and** 2**.

Table 3 shows the concentration (mg/g) of diterpenes** 1** and** 2** in leaves of the investigated* Plectranthus* species. Both compounds were detected in* P. grandis* and* P. barbatus* while* P. ornatus *and* P. amboinicus* showed only the presence of** 2**. The highest concentrations of** 1** and** 2** were found in the leaves of* P. grandis.* Except in this species, diterpene** 2** was found in low concentration in the leaves of the studied species. No amount of** 1** in* P. ornatus *and* P. amboinicus* was observed by the employed methodology.

The lack of clear-cut morphological criteria to discriminate some species of* Plectranthus* has resulted in numerous taxonomic problems for identification of the specimens [1]. Therefore, this work may represent a new tool for discriminating* Plectranthus* species, by using compounds** 1 **and** 2 **as chemomarkers.

## 4. Conclusions

In summary, the analytical methods were adequate for quantification of diterpenes** 1** and** 2** in the leaves of four* Plectranthus* species. Barbatusin (**1**) was identified only in the extracts from* P. grandis *and* P. barbatus*, while compound** 2** was present in all* Plectranthus* species. The highest amount of both compounds** 1** and** 2 **was found in* P. grandis*. This is the first report on the identification of 3*β*-hydroxy-3-deoxybarbatusin (**2**) in the extracts from* P. amboinicus* and* P. ornatus*.

## Figures and Tables

**Table 1:** Analytical parameters of linearity and limits of detection and quantification obtained for **1 **and **2**.

	Analyte	
	**1**	**2**
Regression equation	*y* = 21496*x* − 20448	*y* = 14145*x* + 239256
*r* ^2^	0.9931	0.9966
LOD (*µ*g/mL)	0.015	0.22
LOQ (*µ*g/mL)	0.1	0.6

**Table 2:** Precision and accuracy for **1** and **2** in *Plectranthus *samples.

Precision				Accuracy		
Analyte	Amount *µ*g/mL	Intraday RSD (%)	Interday RSD (%)	Amount added (*µ*g/mL)	Amount found (*µ*g/mL)	Recovery (mean ± RSD, %, *n* = 5)
**1**	85.0	3.42	5.70	186.5	152.3	81.7 ± 1.74
	170.0	2.10	3.31	249.0	259.1	104.0 ± 2.10
	680.0	1.92	6.01	374.0	384.0	102.7 ± 3.39

**2**	65.0	3.44	2.68	37.8	40.3	107.6 ± 3.60
	130.0	3.34	6.21	47.8	50.4	105.4 ± 2.94
	325.0	1.36	10.95	62.8	64.1	102.1 ± 2.73

**Table 3:** Concentration of diterpenes **1** and **2** in leaves of *Plectranthus *species.

Species	**1** (mean ± RSD, mg/g, *n* = 3)	**2** (mean ± RSD, mg/g, *n* = 3)
*P. grandis*	15.432 ± 2.28	4.068 ± 3.34
*P. barbatus*	5.197 ± 3.45	0.654 ± 5.86
*P. ornatus*	Not detected	0.763 ± 5.10
*P. amboinicus*	Not detected	0.160 ± 7.25
